# Supplementary material for: Atomic structure of PI3-kinase SH3 amyloid fibrils by cryo-electron microscopy
Source: Nat Commun. 2019 Aug 21;10:3754. doi: 10.1038/s41467-019-11320-8 (PMC6704188; doi:10.1038/s41467-019-11320-8)
Supplement: Supplementary file 1 — Supplementary Information [file 41467_2019_11320_MOESM1_ESM.pdf]

Supplementary Information

Atomic Structure of PI3-Kinase SH3 Amyloid Fibrils by Cryo-Electron Microscopy

C. Röder *et al.*

Supplementary Table

Supplementary Table 1: Statistics of cryo-EM data collection, reconstruction and model building

**Data Collection**	
Microscope	Tecnai Arctica
Camera	Falcon 3
Acceleration voltage (kV)	200
Nominal Magnification	110,000
Defocus range (µm)	1.5 to 2.25
Dose rate (e^-/ $Å^2$/s)	0.4
Number of movie frames	2520
Exposure time (s)	65
Total electron dose (e^-/$Å^2$)	26.2
Pixel size (Å)	0.935
**Reconstruction**	
Box size (pixel)	200
Interbox distance (Å)	18.8
Number of extracted segments	103,733
Number of segments after 3D classification	27,681
Resolution based on the 0.143 FSC criterion (Å)	3.4
Map sharpening B-Factor ($Å^2$)	150
Helical rise (Å)	2.3548
Helical twist (°)	179.436
Symmetry	C1 (pseudo-2_1 helical)
**Model Composition**	
Non-hydrogen atoms	4494
Number of chains	7
**Model Refinement**	
Resolution (Å)	3.4
Map CC (around atoms)	0.6457
RMSD bonds (Å)	0.008
RMSD angles (°)	1.426
All-atom clash score	6.73
Ramachandran outliers/favored (%)	0/88 %
Rotamer outliers	0/1.59 %
C-beta deviations	0/0
EMRinger score	4.98
Molprobility score	1.97

Supplementary Figures

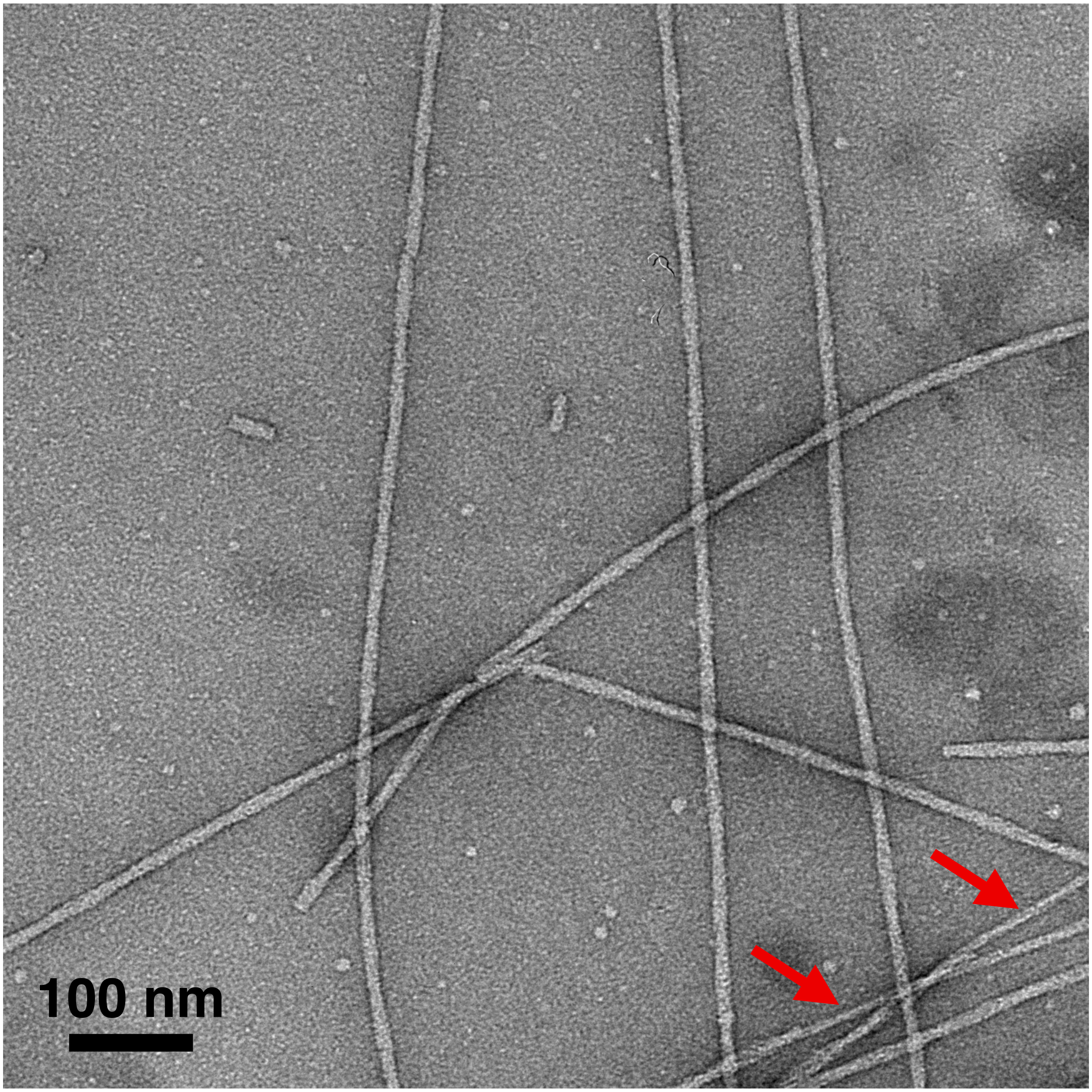

Supplementary Fig. 1 Exemplary negative stain micrograph showing the predominant thick double filament (DF) PI3K-SH3 fibrils and less dominant thin single filament (SF) fibrils (red arrows).

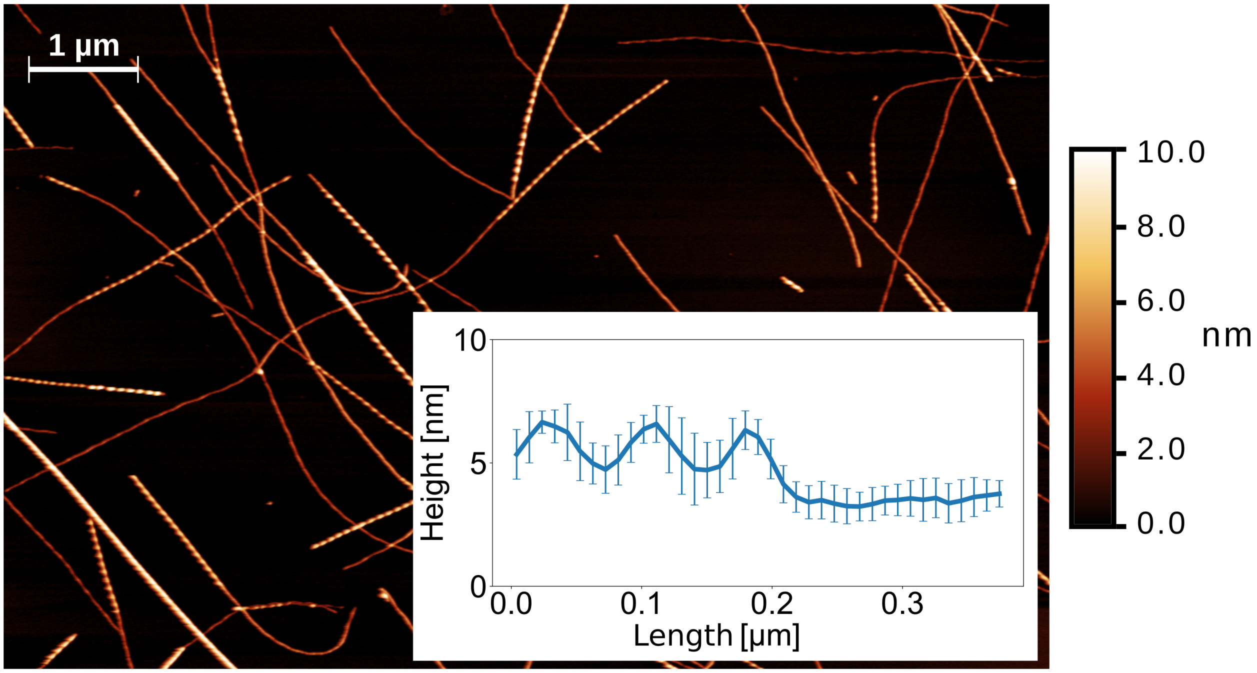

Supplementary Fig. 2 Averaged height pattern of different fibril profiles along their length. The different profiles were aligned on the peak position, the shortest profile was chosen as a minimum length, and their height averaged (error bars correspond to the standard deviation). The first three peaks are indicative of the double filament (DF) portion of the fibrils, while the flat part is indicative of the single filament (SF) portion.

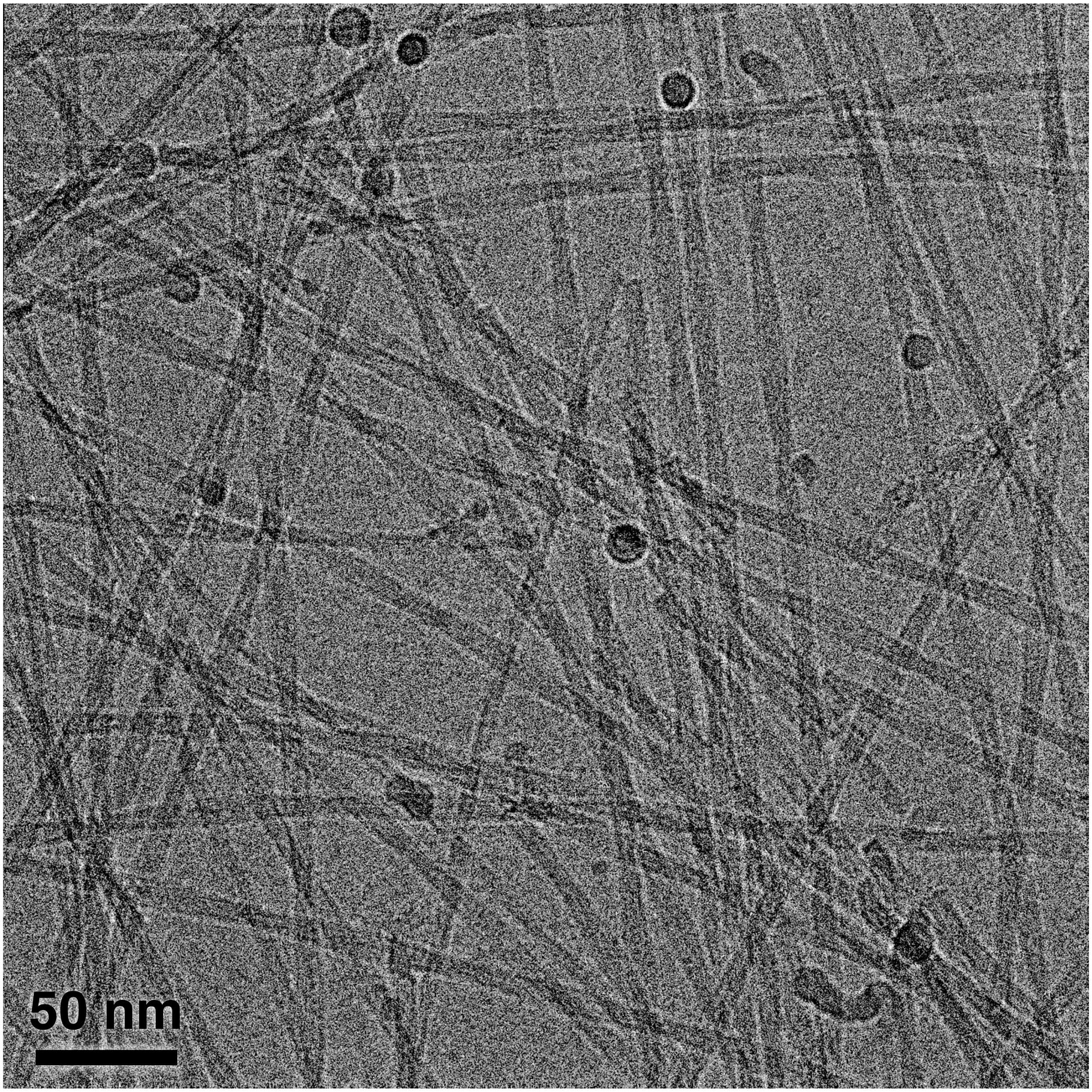

Supplementary Fig. 3 Representative cryo-EM micrograph of DF PI3K-SH3 fibrils. The fibril twist of the thick DF fibril is already well visible in this low-contrast image.

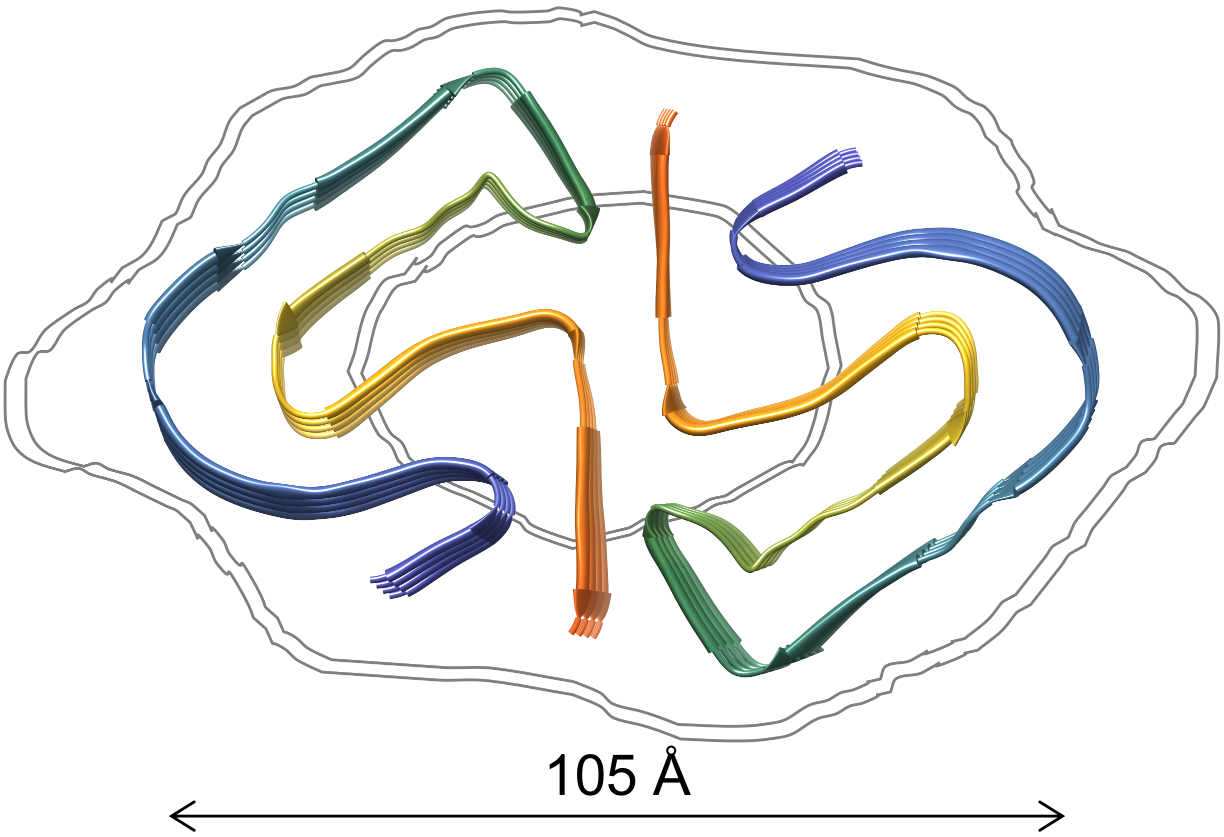

Supplementary Fig. 4 Overlay of the presented DF PI3K-SH3 fibril model with the low-resolution cryo-EM density (contour graphically extracted) from Jimenez *et al.* (1999)[5]. The model and the density are in good agreement, which suggests that both preparations likely yield the same structure.

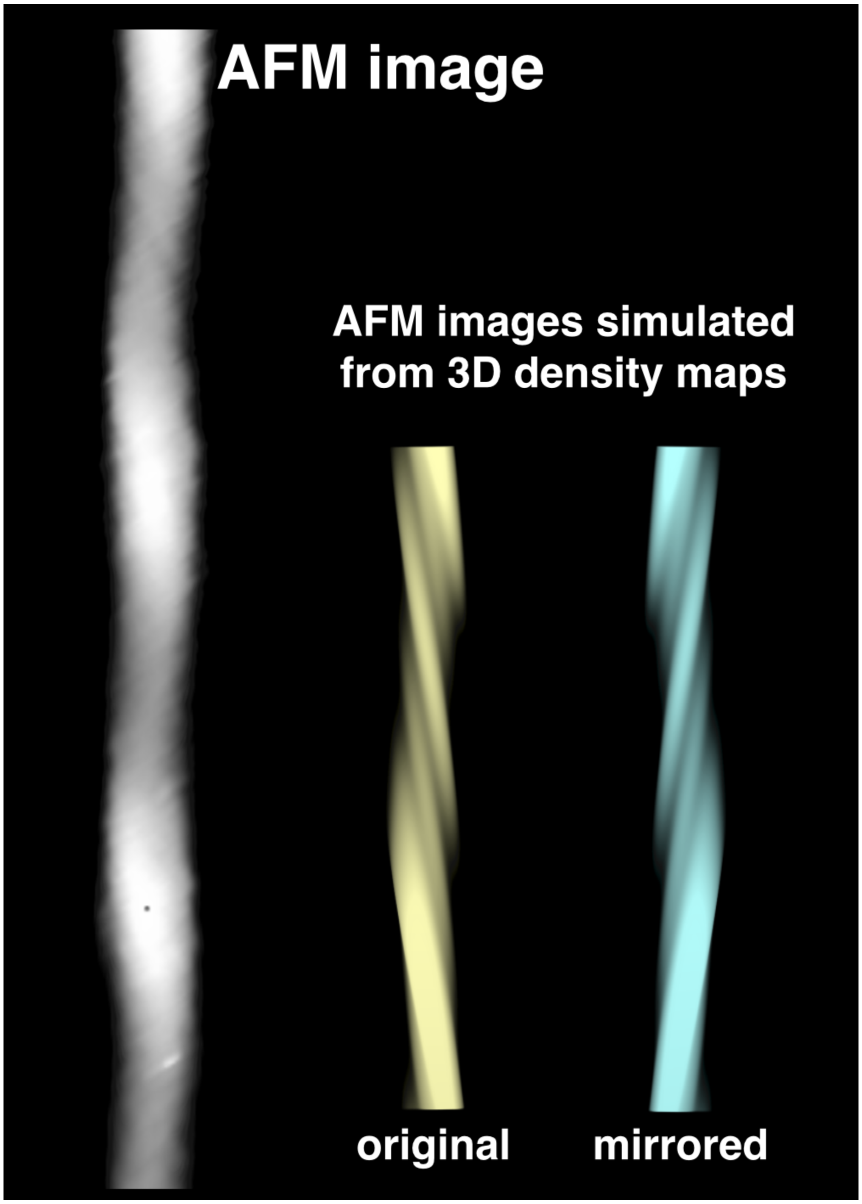

Supplementary Fig. 5 Comparison of an AFM image of a DF PI3K-SH3 fibril with height profiles computed from the 3D EM reconstructions. The original left-handed reconstruction (yellow) yields a higher cross-correlation coefficient (0.943) with the AFM images (grey) than the mirrored reconstruction (0.914, cyan), indicating that the DF PI3K-SH3 fibril is left-handed.

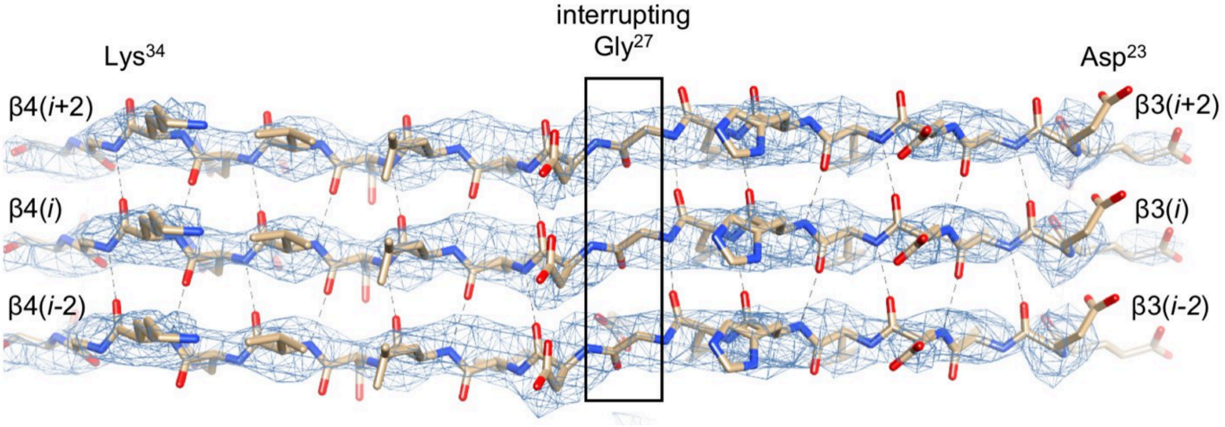

Supplementary Fig. 6 Side view of DF PI3K-SH3 amyloid fibril showing residues 22–35. Displayed are three layers of DF PI3K-SH3 encompassing parts of in-register sheets β3 and β4 with hydrogen bonds highlighted as dashed lines. The cross-β pattern between sheets β3 and β4 is only interrupted by residue Gly^{27}.

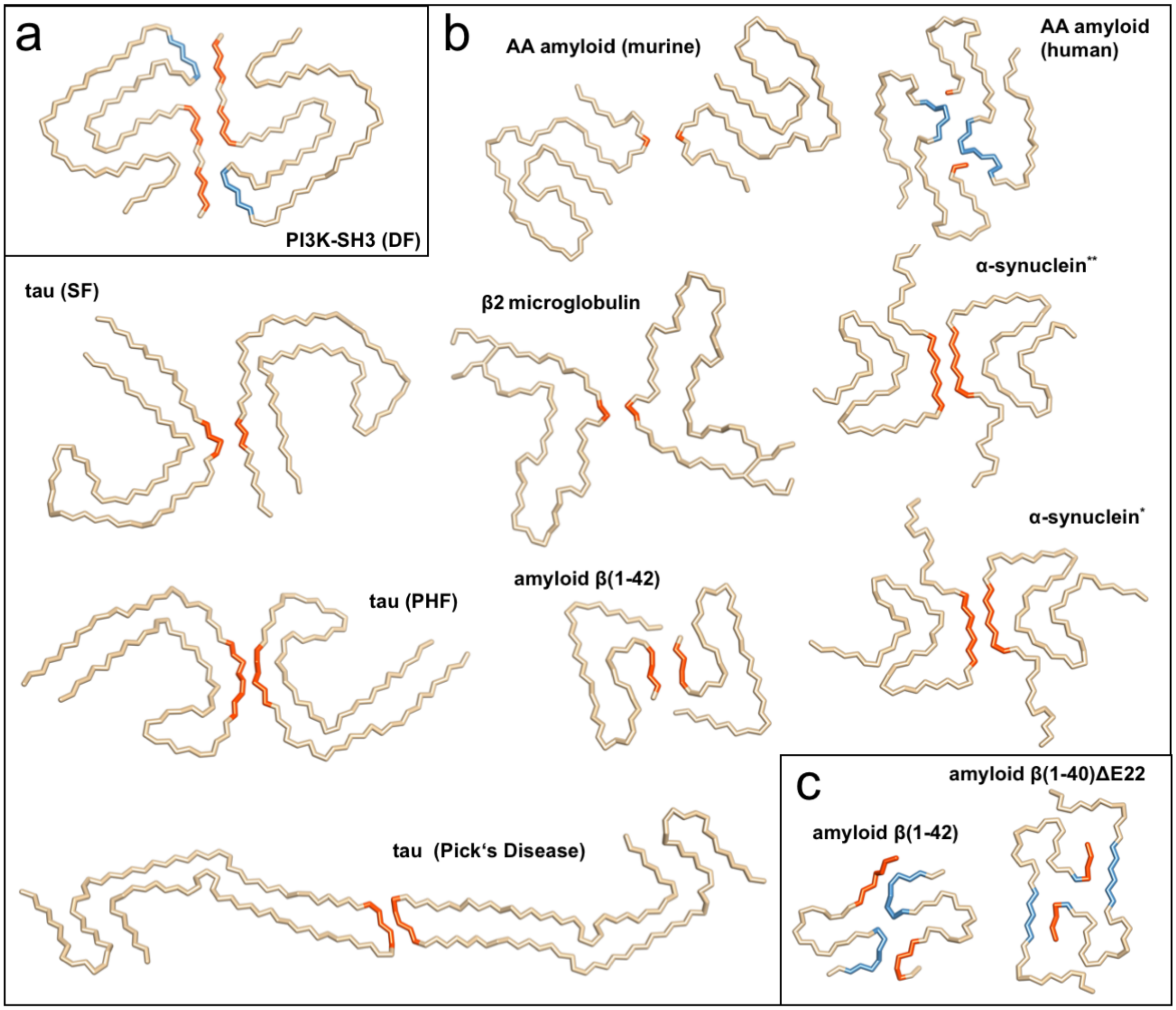

Supplementary Fig. 7 Interface gallery. **a** The protofilament interface of DF PI3K-SH3 fibrils is composed of two sequence regions (orange and blue) and is large compared to those of amyloid fibrils previously determined by cryo-EM (**b**) (Aβ(1–42), PDB: 5OQ5[6]; β2 microglobulin, PDB: 6GK3[7]; AA amyloidosis (human), PDB: 6MST[8]; AA amyloidosis (murine), PDB: 6DSO[8]; α-synuclein*, PDB: 6A6B[9]; α-synuclein**, PDB: 6H6B[10]; tau (PHF)[11], PDB: 5O3L; tau (SF), PDB: 5O3T[11]; tau Pick's Disease, PDB: 6GX5[12]) [6,7,10–13] . **c** Protofilament interfaces of different Aβ variants determined by solid-state NMR (Aβ(1-40)ΔE22, PDB: 2MVX[14]; Aβ(1-42), PDB: 5KK3[15]) exhibit similar sizes and complexities as observed for DF PI3K-SH3 fibrils. Interfaces are shown as Cα-chain. Interface contacts have been defined as Cα-contacts with a cut-off of <10 Å. Beige, no interface

contacts; orange, interface contact formed by first involved β-sheet; blue, interface contact formed by second β-sheet.

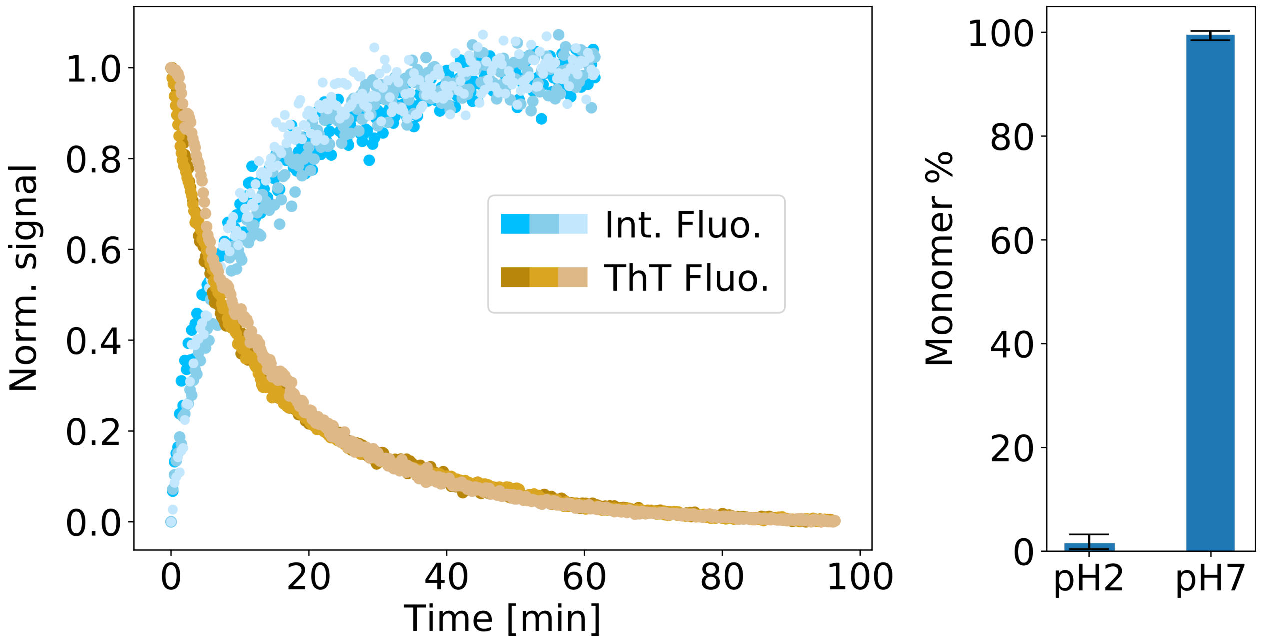

Supplementary Fig. 8 PI3K-SH3 amyloid fibril dissociation at pH 7. Left panel: ThT (brown data points) and intrinsic tryptophan fluorescence intensity ratio 340 nm/310 nm (blue data points) traces of fibril dissociation after dilution into pH 7.4 buffer (measurements are done in triplicate). Right panel: Concentration of protein in the supernatant as percentage of the total protein concentration after incubation for 24 h and centrifugation for 30 min at 16,100 g. Error bars represent the standard deviation on a triplicate measurement. Source data are provided as a Source Data file.

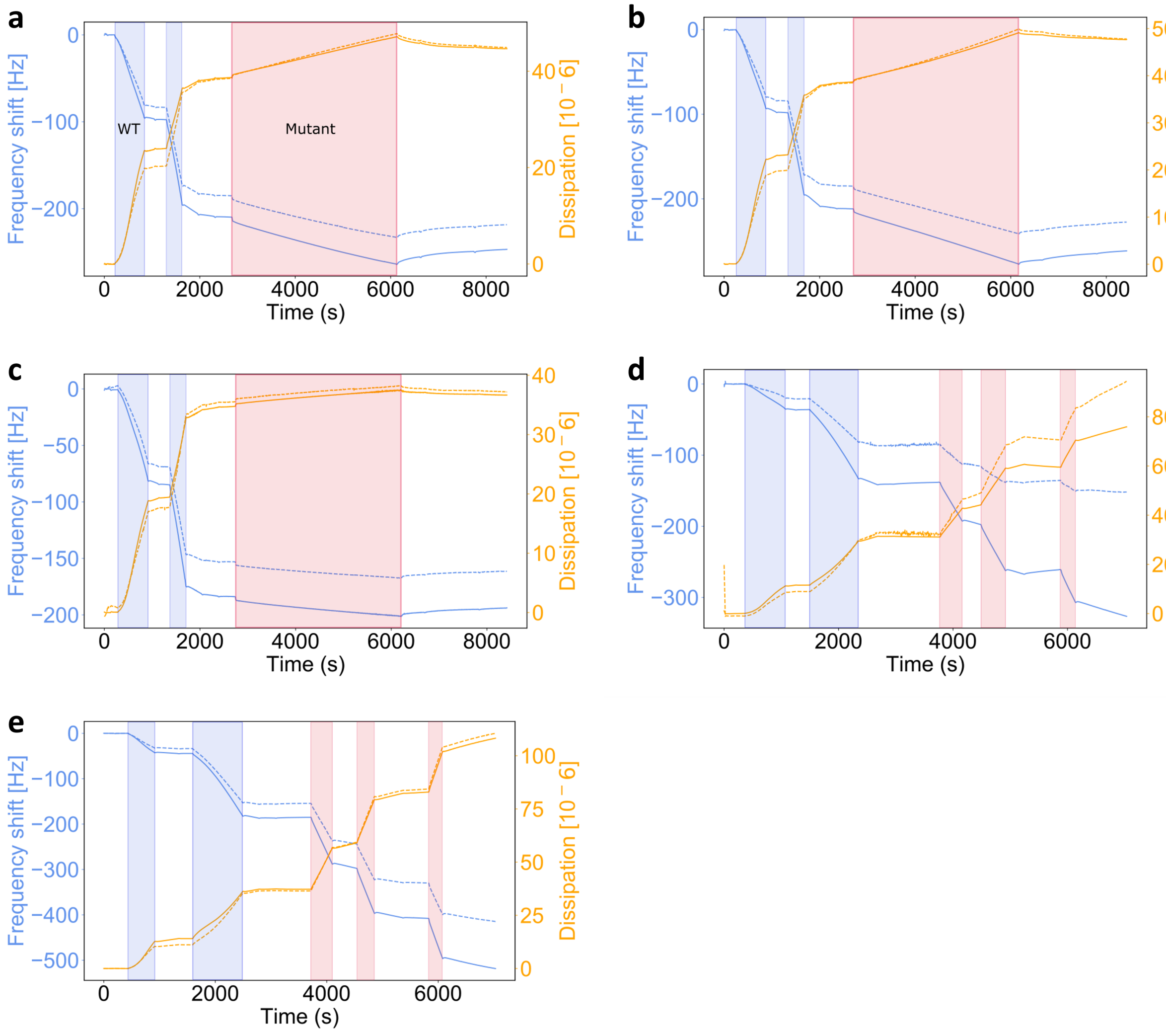

Supplementary Fig. 9 Example of QCM-D traces. Injections of monomeric wild-type (WT) PI3K-SH3 are highlighted with blue bands, and injections of monomeric Ile-to-Ala mutants of PI3K-SH3 are highlighted with red bands (**a** I22A; **b** I29A; **c** I53A; **d** I77A; **e** I82A). The white regions correspond to contact of the QCM sensor surface with buffer. Shown are both the changes in resonant frequency (blue), as well as dissipation (orange). In both cases, the signal for the two overtones N = 3 (dashed line) and N = 5 (solid line) are shown.

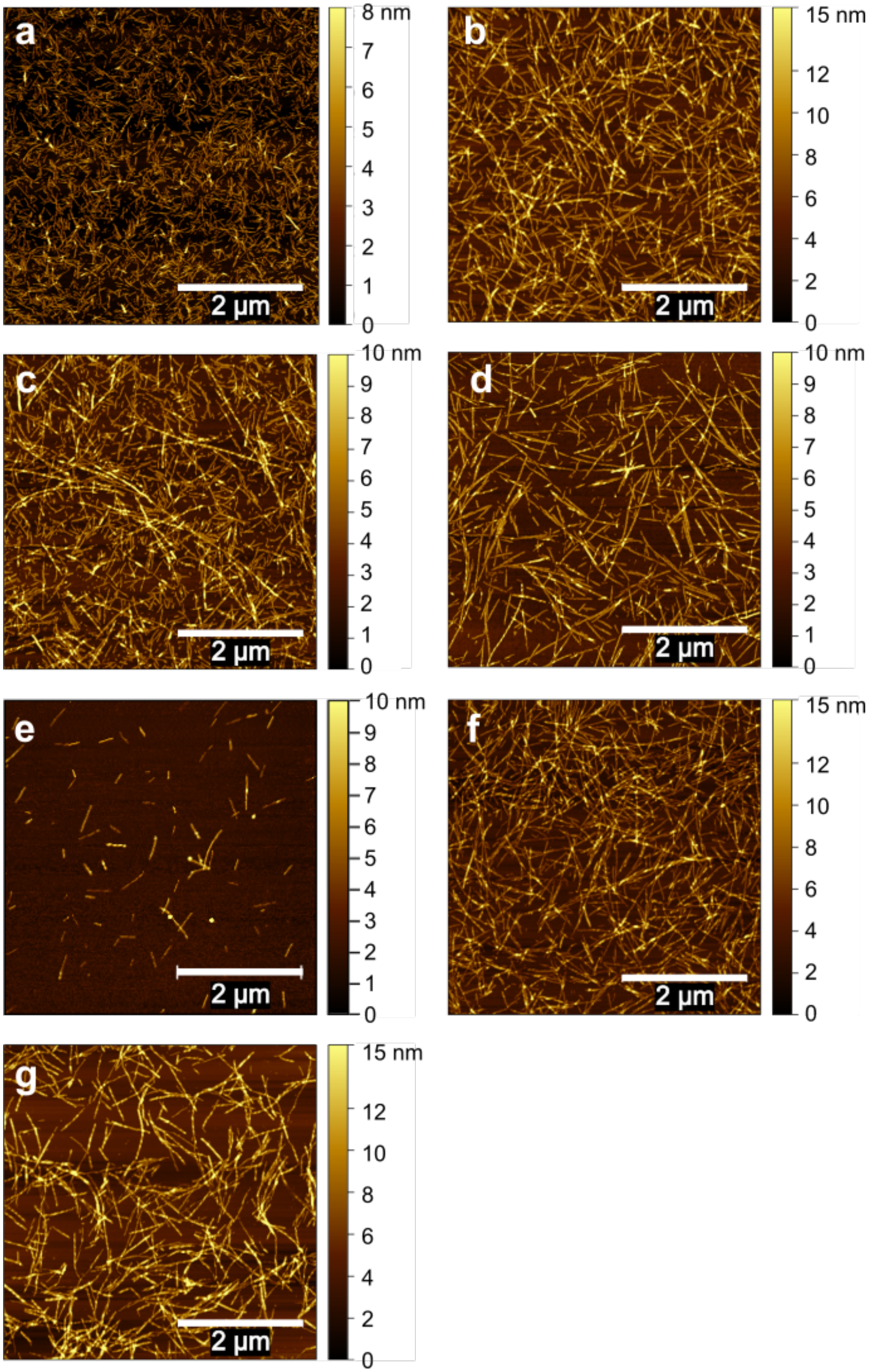

Supplementary Fig. 10 AFM images of **a** WT seeds before elongation experiments. **b** WT seeds incubated with WT monomer. **c**-**g** WT seeds incubated with monomer of **I22A** (**c**), I29A (**d**), I53A (**e**), I77A (**f**), I82A (**g**).

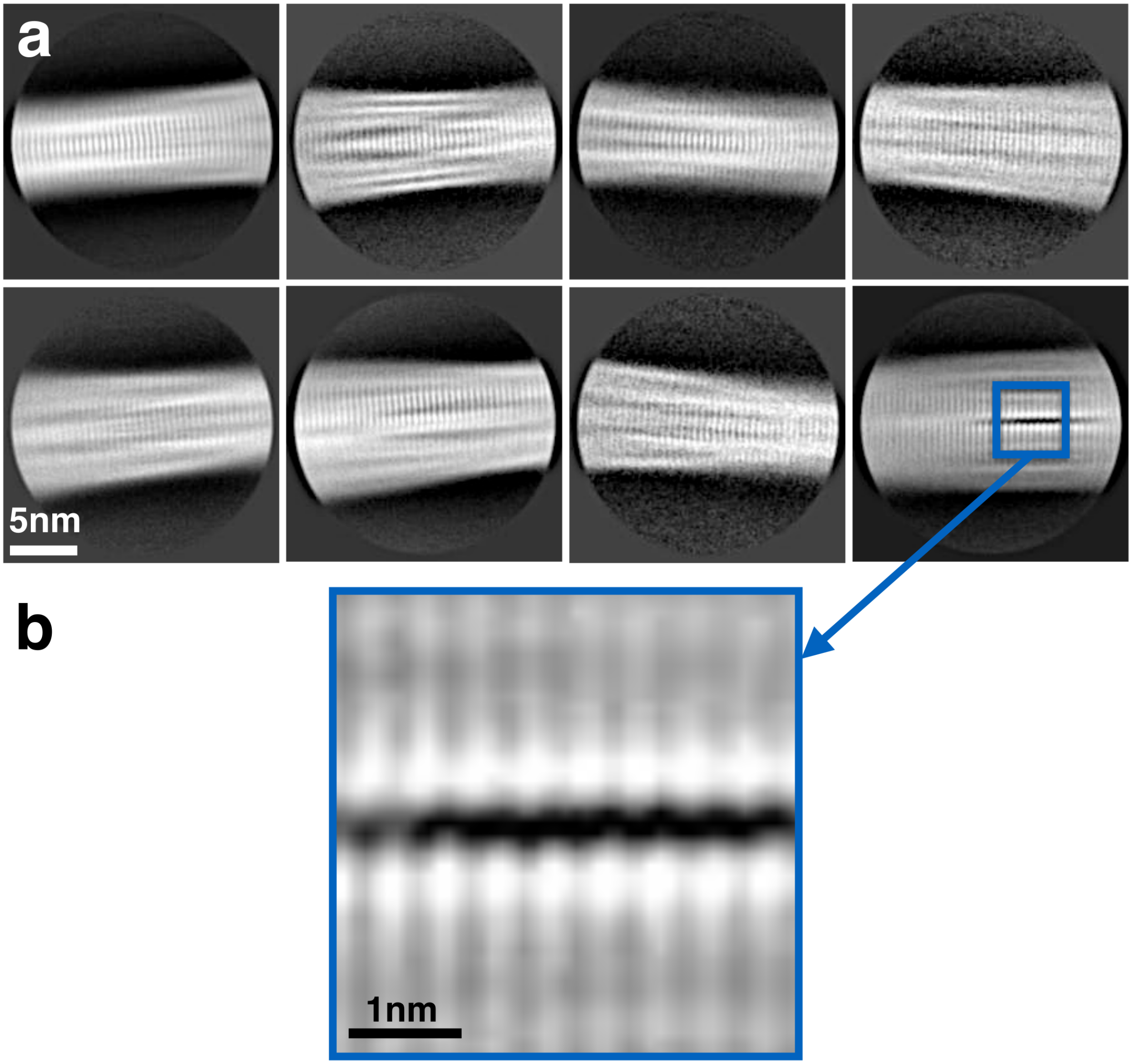

Supplementary Fig. 11 Exemplary 2D classes of DF PI3K-SH3 fibrils. **a** Overview of eight 2D classes comprising 146,215 particles. **b** Magnification of one of the classes displaying the characteristic β-sheet stacking.

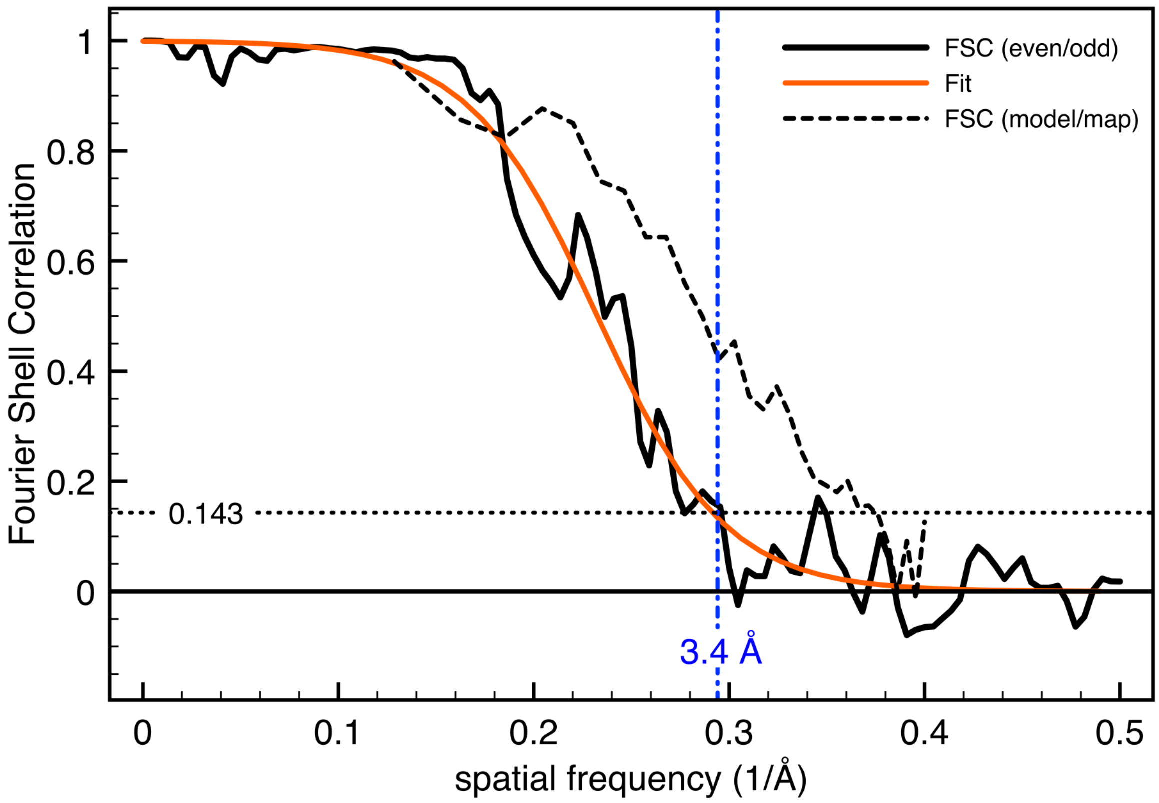

Supplementary Fig. 12 FSC Analysis. FSC curves from the even/odd test (solid black) from the gold-standard refinement yields a resolution of 3.4 Å (using the 0.143 criterion). The even/odd FSC curve is fitted (orange) with the model function 1/(1+e(x-A)/B)) (with A=0.2325 and B=0.03286) to obtain a more robust resolution estimate. The FSC curve comparing the density map computed from the atomic model with the full density reconstruction yields a cross-resolution of 3.4 Å (dashed black).

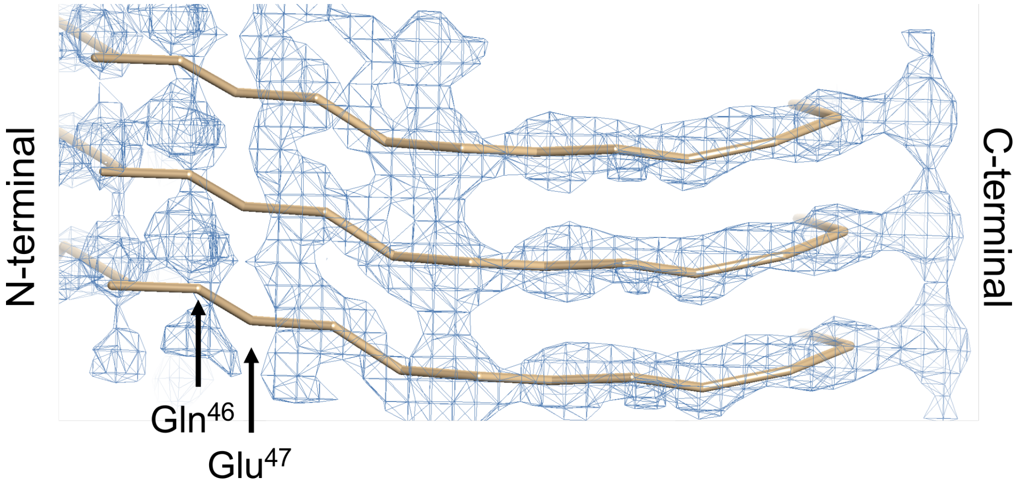

Supplementary Fig. 13 Details of map density between residues 45 and 55. The density around residues Gln^{46} and Glu^{47} does not allow for unambiguous chain tracing, which is mostly due to the weak density of the Glu^{47}. Weak densities of negatively charged residues are typically observed in cryo-EM density maps. The unclear density might also indicate the presence of a less populated second conformer. Detailed magnification of the density map shows the critical area. The Cα-chain trace is shown (brown).

Supplementary References

1. Koyama, S. *et al.* Structure of the PI3K SH3 domain and analysis of the SH3 family. *Cell* **72,** 945–952 (1993).
2. Bayro, M. J. *et al.* High-resolution MAS NMR analysis of PI3-SH3 amyloid fibrils: Backbone conformation and implications for protofilament assembly and structure. *Biochemistry* **49,** 7474–7484 (2010).
3. Kyte, J. & Doolittle, R. F. A simple method for displaying the hydropathic character of a protein. *J. Mol. Biol.* **157,** 105–132 (1982).
4. Buell, A. K. *et al.* Position-dependent electrostatic protection against protein aggregation. *ChemBioChem* **10,** 1309–1312 (2009).
5. Jiménez, J. L. *et al.* Cryo-electron microscopy structure of an SH3 amyloid fibril and model of the molecular packing. *EMBO J.* **18,** 815–821 (1999).
6. Gremer, L. *et al.* Fibril structure of amyloid-β(1–42) by cryo–electron microscopy. *Science* **358,** 116–119 (2017).
7. Iadanza, M. G. *et al.* The structure of a β2-microglobulin fibril suggests a molecular basis for its amyloid polymorphism. *Nat. Commun.* **9,** 4517 (2018).
8. Liberta, F. *et al.* Cryo-EM structure of an amyloid fibril from systemic amyloidosis. *Nat. Commun.* **10,** 1104 (2019).
9. Li, B. *et al.* Cryo-EM of full-length α-synuclein reveals fibril polymorphs with a common structural kernel. *Nat. Commun.* **9,** 3609 (2018).
10. Guerrero-Ferreira, R. *et al.* Cryo-EM structure of alpha-synuclein fibrils. *eLife* **7,** e360402 (2018).
11. Fitzpatrick, A. W. P. *et al.* Cryo-EM structures of tau filaments from Alzheimer's disease. *Nature* **547,** 185–190 (2017).
12. Falcon, B. *et al.* Structures of filaments from Pick's disease reveal a novel tau protein fold. *Nature* **561,** 137–140 (2018).
13. Li, Y. *et al.* Amyloid fibril structure of α-synuclein determined by cryo-electron microscopy. *Cell Res.* **28,** 897–903 (2018).
14. Schütz, A. K. *et al.* Atomic-resolution three-dimensional structure of amyloid β fibrils bearing the osaka mutation. *Angew. Chemie Int. Ed.* **54,** 331–335 (2015).
15. Colvin, M. T. *et al.* Atomic resolution structure of monomorphic Aβ(42) amyloid fibrils. *J. Am. Chem. Soc.* **138,** 9663–9674 (2016).

Primer Sequences

I22A:

GTTTAACTTTAAGAAGGAGATATACCATGGGCAGCAGCCATCACCATCATCATCAT
AGCAGCGGTCTGGTTCCGCGTGGTAGCATGAGTGCCGAAGGTTATCAGTATCGTG
CACTGTATGATTACAAAAAAGAACGCGAAGAAGATGCCGATCTGCACCTGGGTGAT
ATTCTGACCGTTAATAAAGGTAGCCTGGTTGCACTGGGTTTTAGTGATGGTCAAGA
AGCAAAACCGGAAGAAATTGGTTGGCTGAATGGTTATAATGAAACCACCGGTGAAC
GTGGTGATTTTCCGGGTACATATGTTGAATATATCGGTCGCAAAAAAATCAGCCCG
TAGGATCCGGCTGCTAACAAAGCCCGAA

I29A:

GTTTAACTTTAAGAAGGAGATATACCATGGGCAGCAGCCATCACCATCATCATCAT
AGCAGCGGTCTGGTTCCGCGTGGTAGCATGAGTGCCGAAGGTTATCAGTATCGTG
CACTGTATGATTACAAAAAAGAACGCGAAGAGGATATCGATCTGCATCTGGGTGAT
GCACTGACCGTTAATAAAGGTAGCCTGGTTGCACTGGGTTTTAGTGATGGTCAAGA
AGCAAAACCGGAAGAAATTGGTTGGCTGAATGGTTATAATGAAACCACCGGTGAAC
GTGGTGATTTTCCGGGTACATATGTTGAATATATCGGTCGCAAAAAAATCAGCCCG
TAGGATCCGGCTGCTAACAAAGCCCGAA

I53A:

GTTTAACTTTAAGAAGGAGATATACCATGGGCAGCAGCCATCACCATCATCATCAT
AGCAGCGGTCTGGTTCCGCGTGGTAGCATGAGTGCCGAAGGTTATCAGTATCGTG
CACTGTATGATTACAAAAAAGAACGCGAAGAGGATATCGATCTGCATCTGGGTGAT
ATTCTGACCGTTAATAAAGGTAGCCTGGTTGCACTGGGTTTTAGTGATGGTCAAGA
AGCAAAACCGGAAGAGGCAGGTTGGCTGAATGGTTATAATGAAACCACCGGTGAA
CGTGGTGATTTTCCGGGTACATATGTTGAATATATCGGTCGCAAAAAAATCAGCCC
GTAGGATCCGGCTGCTAACAAAGCCCGAA

I77A:

GTTTAACTTTAAGAAGGAGATATACCATGGGCAGCAGCCATCACCATCATCATCAT
AGCAGCGGTCTGGTTCCGCGTGGTAGCATGAGTGCCGAAGGTTATCAGTATCGTG
CACTGTATGATTACAAAAAAGAACGCGAAGAGGATATCGATCTGCATCTGGGTGAT
ATTCTGACCGTTAATAAAGGTAGCCTGGTTGCACTGGGTTTTAGTGATGGTCAAGA
AGCAAAACCGGAAGAAATTGGTTGGCTGAATGGTTATAATGAAACCACCGGTGAAC
GTGGTGATTTTCCGGGTACATATGTTGAATATGCAGGTCGCAAAAAAATCAGCCCG
TAGGATCCGGCTGCTAACAAAGCCCGAA

I82A:

GTTTAACTTTAAGAAGGAGATATACCATGGGCAGCAGCCATCACCATCATCATCAT
AGCAGCGGTCTGGTTCCGCGTGGTAGCATGAGTGCCGAAGGTTATCAGTATCGTG
CACTGTATGATTACAAAAAAGAACGCGAAGAGGATATCGATCTGCATCTGGGTGAT
ATTCTGACCGTTAATAAAGGTAGCCTGGTTGCACTGGGTTTTAGTGATGGTCAAGA
AGCAAAACCGGAAGAAATTGGTTGGCTGAATGGTTATAATGAAACCACCGGTGAAC
GTGGTGATTTTCCGGGTACATATGTTGAATATATCGGTCGTAAAAAAGCGAGCCCG
TAGGATCCGGCTGCTAACAAAGCCCGAA
